# Variations in microbial community structure and functional gene expression in bio-treatment processes with odorous pollutants

**DOI:** 10.1038/s41598-019-54281-0

**Published:** 2019-11-28

**Authors:** Weidong Li, Jianguo Ni, Shaoqin Cai, Ying Liu, Chenjia Shen, Huayun Yang, Yuquan Chen, Jia Tao, Yunfeng Yu, Qi Liu

**Affiliations:** 1 College of Qianjiang, Hangzhou Normal University, Hangzhou, 310036 Zhejiang People’s Republic of China; 2 Hangzhou Ecological Environment Bureau of Xiaoshan Branch, Hangzhou, 311201 Zhejiang People’s Republic of China; 3 College of Life and Environmental Science, Hangzhou Normal University, Hangzhou, 310036 Zhejiang People’s Republic of China; 4 College of Environment, Zhejiang University of Technology, Hangzhou, 310014 Zhejiang People’s Republic of China

**Keywords:** Pollution remediation, Natural hazards

## Abstract

Engineered microbial ecosystems in biofilters have been widely applied to treat odorous gases from industrial emissions. Variations in microbial community structure and function associated with the removal of odorous gases by biofilters are largely unknown. This study performed a metagenomic analysis to discover shifts in microbial community structures in a commercial scale biofilter after treating odorous gas. Our study identified 175,675 functional genes assigned into 43 functional KEGG pathways. Based on the unigene sequences, there were significant changes in microbial community structures in the biofilter after treating odorous gas. The dominant genera were *Thiobacillus* and *Oceanicaulis* before the treatment, and were *Acidithiobacillus* and *Ferroplasma* after the treatment. A clustering analysis showed that the number of down-regulated microbes exceeded the number of up-regulated microbes, suggesting that odorous gas treatment reduced in microbial community structures. A differential expression analysis identified 29,975 up- and 452,599 down-regulated genes. An enrichment analysis showed 17 classic types of xenobiotic biodegradation pathways. The results identified 16 and 15 genes involved in ammonia and sulfite metabolism, respectively; an analysis of their relative abundance identified several up-regulated genes, which may be efficient genes involved in removing odorous gases. The data provided in this study demonstrate the changes in microbial communities and help identify the dominant microflora and genes that play key roles in treating odorous gases.

## Introduction

Odorous gases from industrial emissions significantly contribute to haze pollution and photochemical contamination in China^1^. Odorous pollutants, including major and typical outdoor air pollutants, threaten personal health and deteriorate public welfare^2^. Odors generally consist of ammonia and sulfur compounds; and complex odors have detrimental effects on ecological and environmental functions^3,4^. Thus, reducing odorous emission sources is an effective strategy for controlling photochemical air pollution^5^.

The national emission standards of odorous has be updated recently, requiring stricter emission standards (DB33-2146-2018) than before. Many treatment methods, including physical, chemical, and biological technologies, have been developed to limit odorous gas emissions^6^. Physical-chemical methods effectively treat odorous gases; however, their application depend on having high concentrations, come at a high cost, and create secondary pollutions^7^. Several biotechnologies, including biofilters, bioscrubbers, and biotrickling filters, are currently considered the most promising and environment-friendly approaches for treating low concentrations of odorous gases^8^. Due to differences in the microbial community and liquid states, different bioreactors are used to treat different kinds of pollutants^2^. Biofiltration has become a viable and economic technology to treat industrial emissions containing low concentrations of odorous gases^9^.

Degrading pollutants into smaller, less harmful molecules depends on the capacity of a highly effective microbial community^10^. Recently, there have been more studies on the structural and functional responses of microbial communities when treating odorous gases. For example, reactors supplied with a gaseous effluent resulted in the simplification of the structure of a bacterial community and the appearance of a dominant microflora^11^. Another study investigated the correlations between odor and microbial composition during food waste composting^12^. Analyzing microbial communities is a shortcut to screen high-efficiency strain and optimize a biofiltration system.

Microbial communities play essential roles in engineered microbial ecosystems^13^. Research has identified a number of waste gas catabolic microbial strains^13^. For example, studies have investigated the role of *Sphingomonas* sp. “D3K1” in degrading ethylbenzene; *Thiomonas* sp. “WZW” in degrading carbon disulfide; and *Acidithiobacillus thiooxidans* in degrading hydrogen sulfide^14–16^. High-throughput sequencing has shown to be effective in revealing variations in microbial communities^17^. To uncover the microbial characteristics in bioreactors, previous studies have also developed new metagenomic sequencing techniques^18,19^. However, how complex gases containing H_2_S and NH_3_ effect the microbial community structure in a biofilter system are largely unknown.

This study applied high-throughput metagenomics sequencing to investigate how microbial compositions and gene transcript profiles respond to the biodegradation of odorous gases in a biofilter system. Several target strains and functional genes will be used for improving the efficiency of biofilter. The data provided by this study demonstrates the changes in the microbial communities and helps identify the dominant microflora that play key roles in treating odorous gases.

## Materials and Methods

### Materials and sampling

**Figure 1:**
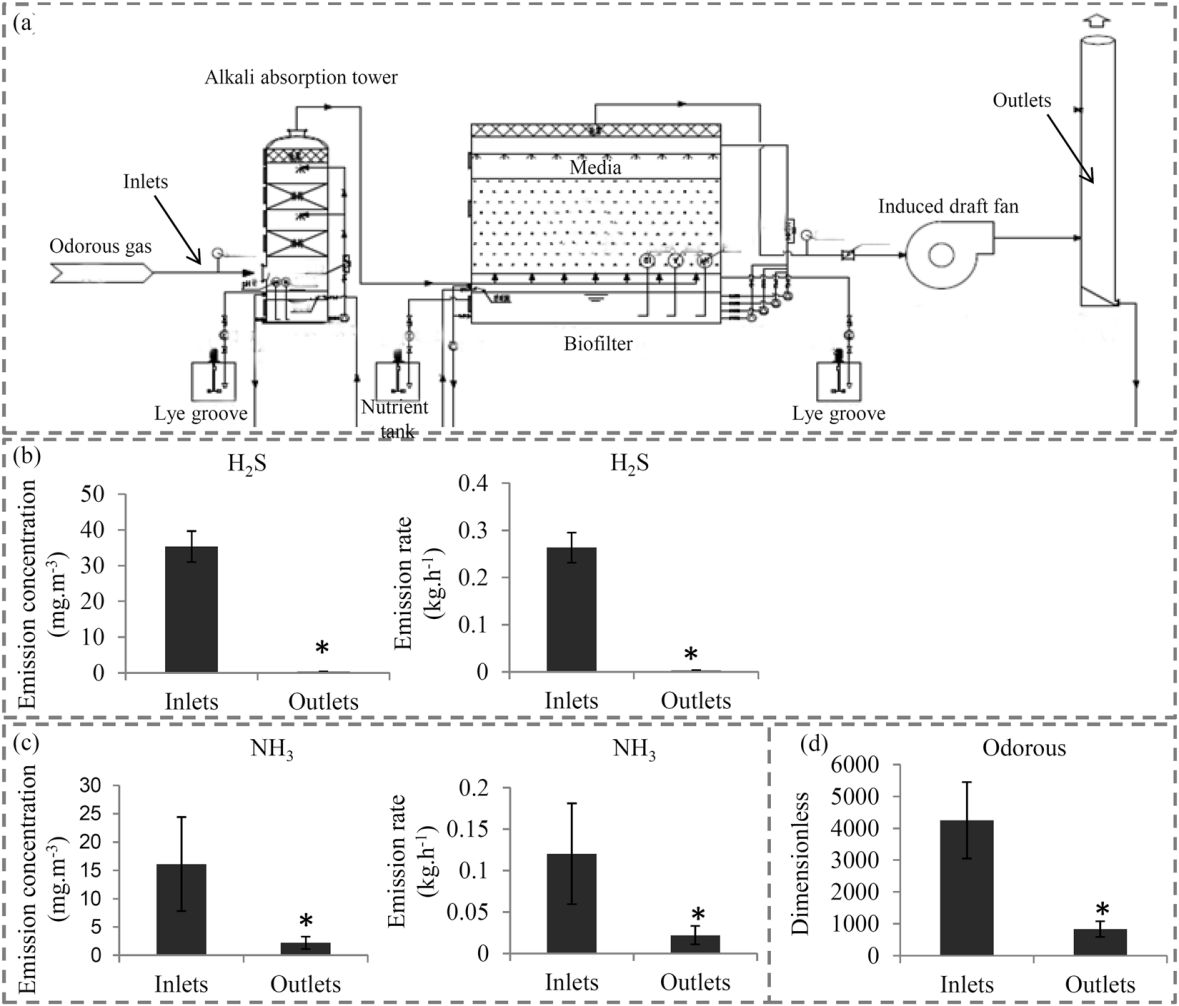
Removal performance for odorous gases of our biofilter construction. (**a**) A sketch map showed the details of the biofilter construction in our study. (**b**) The emission concentrations and emission rates of H2S in the inlet and outlet of the biofilter construction. (**c**) The emission concentrations and emission rates of NH3 in the inlet and outlet of the biofilter construction. (**d**) The dimensionless of odorous gases in the inlet and outlet of the biofilter construction. The significant variations (p < 0.05between the inlet and outlet were indicated by “*”. Error bars represent mean ± SD (n = 3).

Mixed media, containing activated carbon, wheat bran, and sawdust (1:1:2), was used to biodegrade the odorous gases. Sample materials were enriched using activated sludge from a waste gas treatment plant (Wenzhou, China); there was a 3-week acclimation period with continuous clean air (Before Treatment, BT), followed by a week period of continuous odor-contaminated airflow (After Treatment, AT). The main components of odorous gases are H_2_S and NH_3._ The initial concentrations of H_2_S and NH_3_ are 35.3 mg.m^−3^ and 16.1 mg.m^−3^, respectively. The emission rate of H_2_S and NH_3_ are 0.26 kg.h^−1^ and 0.26 kg.h^−1^, respectively. The contaminated air passed through the media in an up-flow direction. Figure 1a shows the details of the biofilter construction.

### Chemical analytical methods

The odor was determined using the classical a sensory test method termed the triangle-odor-bag method^20^. In detail, five sniffers were employed as a panel group. During the determination, two vacuum bags were filled with gases from the biofilter’s inlets and outlets using a vacuum pump. If the sniffer identified the smelly bags correctly, the smelly bags were diluted and sniffed again. The experiment was stopped until the odour concentration of the diluted sample was lower than the olfactory threshold of the sniffer. Each sample was measured by five sniffers at the same time. Finally, the odour concentration was obtained according to the individual threshold of the sniffer and the average threshold of the sniffing group. The odor index was defined using a series of formula described by Noguchi’s work^21^. The NH_3_ concentrations were measured using the Nessler’s reagent colorimetry method^22^. The H_2_S concentrations were measured using the methylene blue spectrophometry method (GB/T11742-89, China).

### DNA isolation

The microbial samples were extracted from the media of biofilter. For the BT group, the sample was extracted after a 3-week acclimation period with continuous clean air. After a 3-week acclimation, the sample of AT group was extracted after a week period of continuous odor-contaminated airflow. Total DNAs were isolated from 10.0 mL sample aliquots using a DNA kit (D4015-02, Omega, Inc., USA) following the kit’s procedures. The reagent was added to obtain trace DNA from each sample, and was effectively used to prepare the DNA of most microbes. Control samples consisting of blank swabs were treated through the DNA kit to confirm they contain no DNA amplifications. DNAs were eluted in a 50 μL elution buffer according to the manufacturer’s method (QIAGEN). The samples were stored at −80 °C until used by LC-BIO TECHNOLOGIES (Hangzhou) CO., LTD. China.

### Preparation of DNA libraries

Two sequencing libraries were developed to refer to the digested sludge sample before treatment (BT) and after treatment (AT). These were constructed using 5 μg of genomic DNA. Preparation of DNA libraries were performed according to the previous published work^23^. The DNA libraries were constructed using the Nano DNA Library Preparation Kit (FC-121-4001). The DNA was fragmented using NEB dsDNA Fragmentase during a 30-min incubation period at 37 °C. Construction of DNA library start with DNA fragments. Blunt-end fragments were produced using a fill-in reactions. Fragment sizes were selected using sample purification beads. A-bases were added to the blunt ends of strands, ensuring them to be ligated to the adapters. Each adapter contained a T-base overhang for ligating the adapter to the A-tailed DNA fragments. The adapter contains the complement of sequencing primer hybridization sites for single, paired-end, and indexed reads. Single- or dual-index adapters were added to the DNA fragments and the final products were amplified using the following PCR conditions: an initial denaturation at 95 °C for 3 min; 8 cycles of denaturation at 98 °C for 15 s, annealing at 60 °C for 15 s, and extension at 72 °C for 30 s; and then final extension at 72 °C for 5 min.

### Illumina sequencing and quality control

High-throughput sequencing was conducted on a HiSeq4000 platform, with the sequencing mode set at PE150. Raw sequences were treated to get valid reads. Adapters were removed from raw reads using software Cutadapt v1.9. Then, low quality reads were processed using Fqtrim v0.94 with a sliding-window algorithm. The raw sequence data has been submitted to the NCBI as a BioProject with accession number PRJNA522625.

### *De novo* assembly and unigene analysis

*De novo* assembly and unigene analysis were performed according to the previous published work^24^. The clean reads were *de novo* assembled to produce the metagenome using software SPAdes v3.10.0. All coding sequences (CDS) of the metagenomic contigs were predicted using software MetaGeneMark v3.26. The CDSs of all sample groups were clustered by software CD-HIT v4.6.1 to get unigenes. The abundance of unigene in each sample were evaluated by Transcripts Per Million (TPM) based on the number of aligned reads using bowtie2 v2.2.0. The lowest common ancestor taxonomy of unigenes were got by aligning unigene sequences against the NR, GO, KEGG, CAZy, CARD and PHI databases by software DIAMOND v. 0.7.12 with default parameters. Based on the taxonomic and functional annotation of unigenes, along with the abundance profile of unigenes, the differential analysis were conducted at each taxonomic or functional or gene-wise level, using the Kruskal-Wallis test.

### Analysis of taxonomic profile

The unigenes were searched against the Nr_meta database (blastp, evalue <1e^−5^) using the DIAMOND software, and the results with evalue <1e^−10^ were selected for species classification. Applying the NCBI Taxonomy system, each classification level of the sequence was obtained using a LCA algorithm using MEGAN software, including the SuperKingdom, Phylum, Class, Order, and Family levels. The diversity of microbial communities in the BT and AT samples was evaluated using the Chao1 richness index and Shannon diversity index from the unigene data at different classification levels^25^.

### Statistical analysis

Three parallel experiments were carried out for sequencing. All data on the diversity indexes, the relative abundances of unigenes, and the H_2_S and NH_3_ levels were processed using SPSS 18.0. One-way analysis of variance (ANOVA) was applied to analyze the differences between the BT and AT sample groups. Wilcoxon tests were conducted to detect differences in microbial community structures between two groups. The *P* value was produced by the false discovery rate (FDR) analysis and adjusted using the Benjamini and Hochberg’s method^26^. A significant difference was indicated by a probability value (*P*) less than 0.05.

## Results

### Removal performance for odorous gases

The study evaluated biofilter performance in removing complex mixture gases containing H_2_S, NH_3,_ and other odorous gases. After treatment, the emission concentrations of H_2_S decreased from 35.3 mg.m^−3^ to 0.32 mg.m^−3^; the emission rate of H_2_S were reduced from 0.26 kg.h^−1^ to 0.0032 kg.h^−1^ (Fig. 1b). After treatment, emission concentrations of NH_3_ decreased from 16.1 mg.m^−3^ to 2.2 mg.m^−3^; the emission rate of NH_3_ were reduced from 0.26 kg.h^−1^ to 0.0032 kg.h^−1^ (Fig. 1c). The biofilter absorbed 80.4% of odorous material as a result of the treatment (Fig. 1d).

### Overview of the metagenomes

A total of 1.8E + 08 and 1.6E + 08 raw reads were obtained from the BT and AT samples, respectively. After filtering, 3.2E + 08 clean reads were produced to assemble the metagenome (Table S1). Pair-wise Pearson’s correlation coefficients of the three replicates × two sample groups indicated the sequencing data had good repeatability (Fig. S1). The coding region of all assembled contigs was predicted to be 496,718 open reading frames (ORFs), with an average length of 576 bp.

### Functional gene prediction and classification

**Figure 2:**
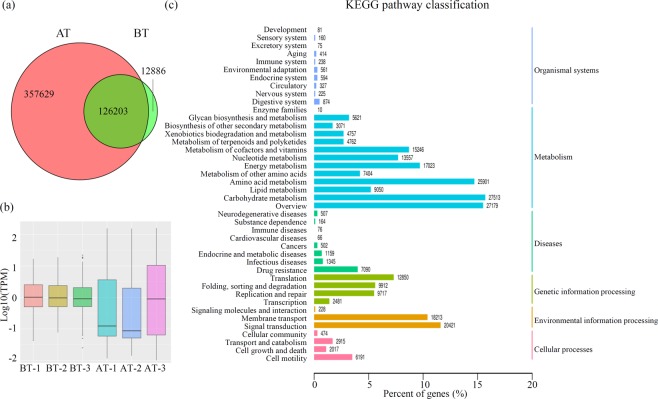
Overview of the metagenomes. (**a**) The numbers of unigene identified in the BT and AT sample groups are shown in a Venn diagram. Red indicated the unigenes in the AT sample group and green indicated the unigenes in the BT sample group. (**b**) The box-plot analysis showed that ranges of the expression abundances of unigenes from both of the BT and AT sample groups. (**c**) The KEGG functional prediction and classification of all identified unigenes.

In total, the study identified 126,203 common unigenes, 357,629 AT-specific unigenes, and 12,886 BT-specific unigenes (Fig. 2a). Figure S2a shows the length distribution of all predicted unigenes; Fig. S2b shows the densities of unigene expression in each sample. The box-plot analysis shows that the ranges of the expression abundances of unigenes from the AT group exceeded the ranges from the BT group (Fig. 2b).

Most unigenes were mapped to different KEGG pathways. In total, 175,675 unigenes were assigned to 43 functional KEGG pathways. In the ‘organismal systems’ category, the most represented KEGG pathways were the ‘digestive system’ (874 unigenes), ‘endocrine system’ (594 unigenes) and ‘environmental adaptation’ (561 unigenes). In the ‘metabolism’ category, the most represented KEGG pathways were ‘carbohydrate metabolism’ (27,513 unigenes), ‘amino acid metabolism’ (25,901 unigenes), and ‘energy metabolism’ (17,023 unigenes). In the ‘diseases’ category, the largest KEGG term was ‘drug resistance’ (7,090 unigenes). In the ‘genetic information processing’ category, most unigenes were grouped into the ‘translation’ (12,850 unigenes), ‘folding, sorting and degradation’ (9,912 unigenes), and ‘replication and repair’ (9,717 unigenes) terms. In the ‘environmental information processing’ category, most unigenes were grouped into the ‘membrane transport’ (18,213 unigenes) and ‘signal transduction’ (20,421 unigenes). In the ‘cellular processes’ category, the largest KEGG term was ‘cell motility’ (6,191 unigenes) (Fig. 2c).

### Comparative taxonomic profile of BT and AT metagenomes

**Figure 3:**
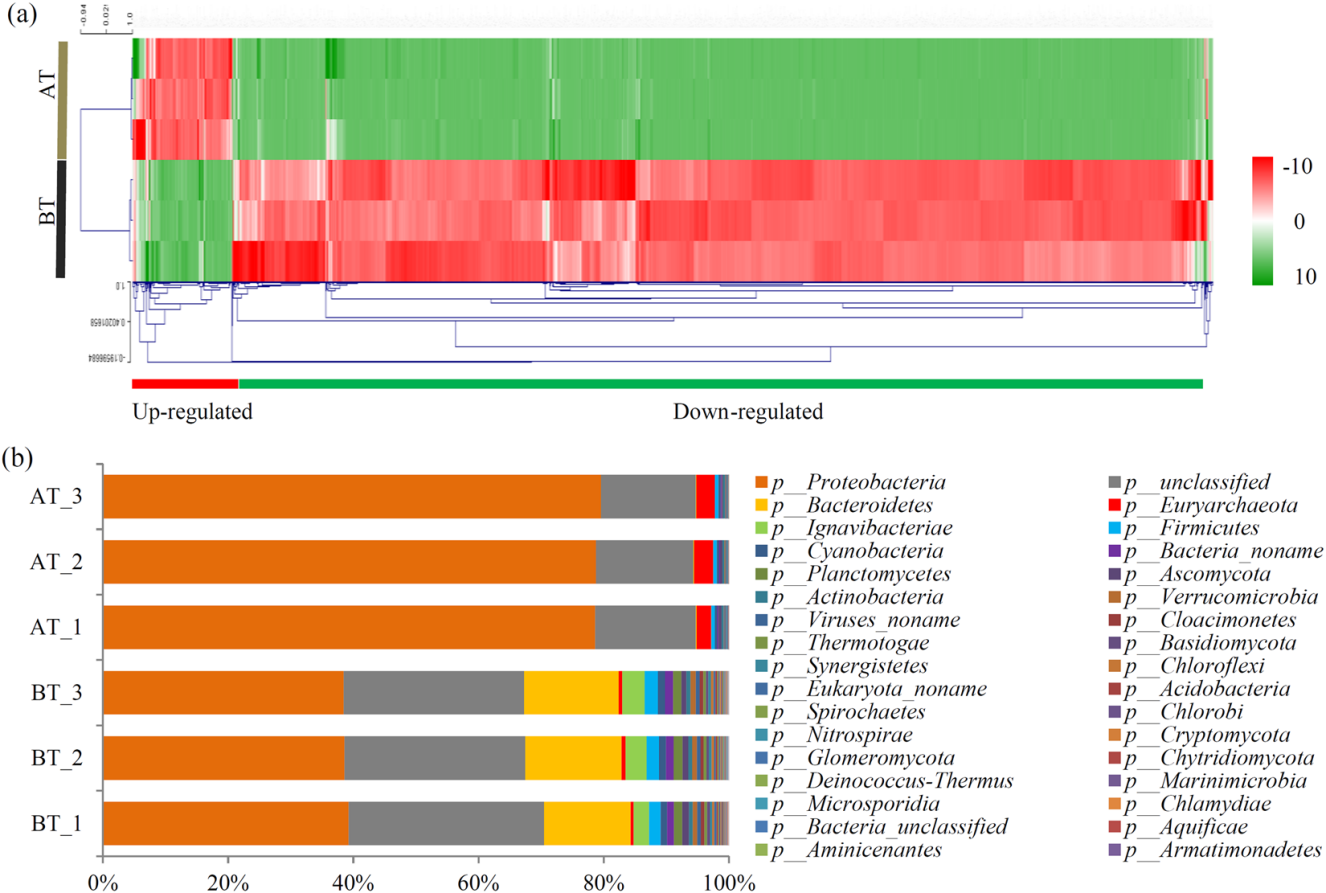
Comparative taxonomic profile of BT and AT metagenomes. (**a**) Microbial compositions for both BT and AT sample groups at genus level. Color intensity in each panel shows the relative abundances of each representative species in the BT and AT sample groups. (**b**) Microbial compositions for both BT and AT sample groups at phylum level. The scale unit of the heatmap is RPKM (Reads Per Kilobase per Million mapped reads). The heatmap scale ranges from −10 to +10 on a log_2_ (RPKM).

To generate microbial information, 496,718 unigene sequences were used for the taxonomic and functional analyses (Table S2). Significant differences in microbial community structures were observed between the BT and AT sample groups. A clustering analysis showed that the number of down-regulated microbes exceeded the number of up-regulated microbes (Fig. 3a).

At the phylum level, the major phyla were *Proteobacteria*, *Bacteroidetes*, and *Ignavibacteriae*. The proportions of *Proteobacteria* and *Euryarchaeota* increased significantly, from 38.76% to 78.99% and from 0.57% to 2.77%, respectively. Additionally, the proportions of *Nitrospirae* and *Parvarchaeota* were also significantly up-regulated after treatment. The proportion of other phyla significantly decreased after treatment, as follows: *Bacteroidetes* (from 14.77% to 0.11%), *Ignavibacteriae* (from 3.15% to 0.012%), *Firmicutes* (1.97% to 0.55%), and *Cyanobacteria* (1.1% to 0.38%) (Fig. 3b and Table S3). The major genera were *Thiobacillus* (0.51%) and *Oceanicaulis* (0.27%) in the BT samples; the major genera were *Acidithiobacillus* (70.1%) and *Ferroplasma* (2.58%) in the AT samples. At the genus level, 7 genera were only detected in the AT samples and 254 genera were only detected in the BT samples (Table S4).

### Analysis of DEGs

**Figure 4:**
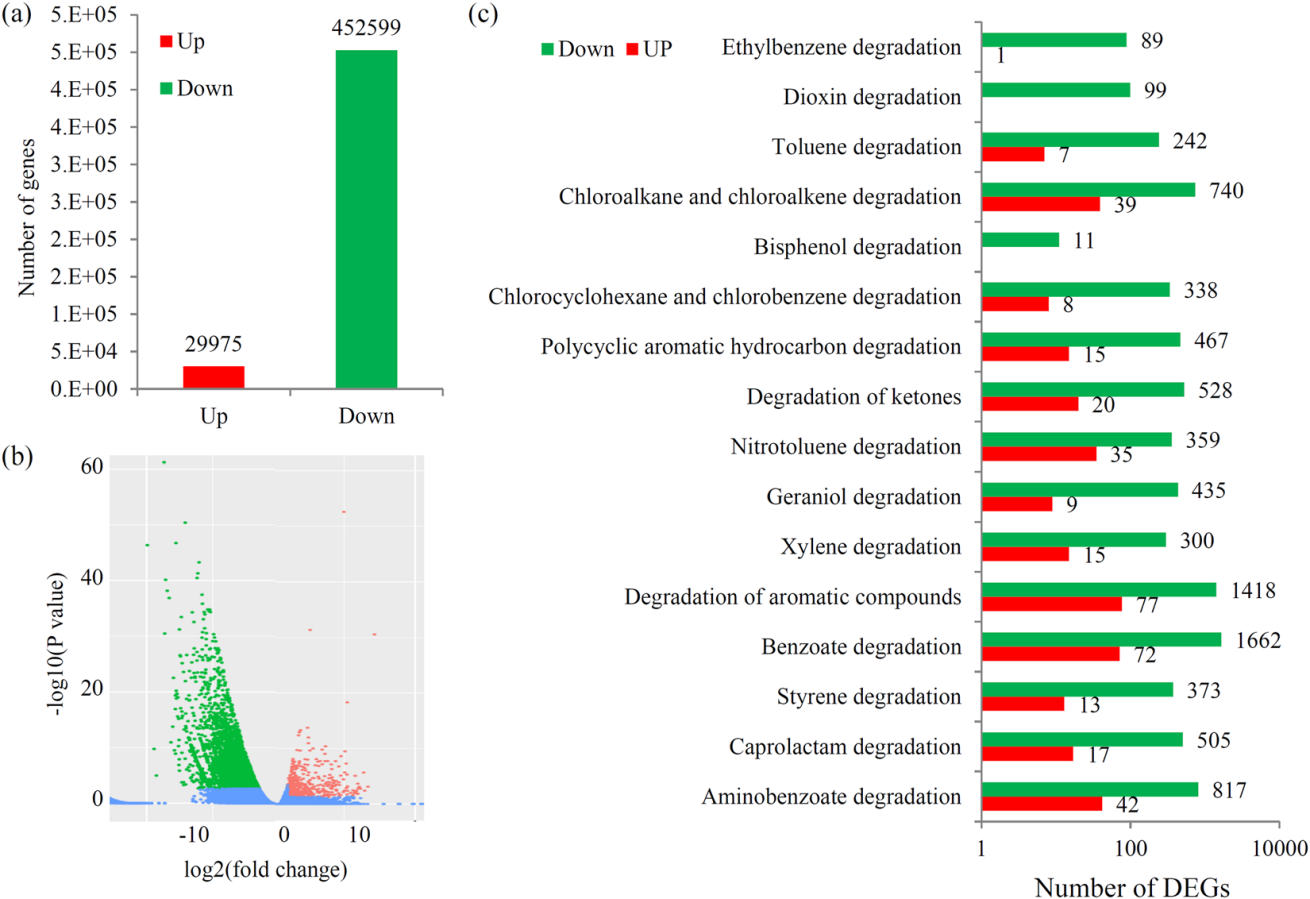
Analysis of differential expressed genes (DEGs). (**a**) The number of up- and down-regulated genes after treatment. (**b**) Significance analysis of the DEGs between the AT and BT sample groups by Volcanoplot. (**c**) The number of up- and down-regulated genes related to xenobiotic biodegradation pathways after treatment.

The study identified a large number of DEGs, including 29,975 up-regulated and 452,599 down-regulated genes (Fig. 4a and Table S5). A significance analysis of the DEGs between the AT and BT sample groups was visualized using a volcano plot (Fig. 4b). Among these DEGs, most unigenes were annotated from sequence-based homologies. For the GO classification, the top three largest GO terms in biological process were “biological process”, “proteolysis”, and “DNA replication.” In the cellular component, the top three largest GO terms were “cellular component”, “outer membrance-bounded periplasmic apace”, and “cell outer membrance.” In the molecular function, the three largest GO terms were “molecular function”, “transporter activity”, and “phosphorelay sensor kinase activity” (Fig. S3).

### Enrichment analysis of the xenobiotic biodegradation pathways

All the DEGs were assigned into 387 KEGG metabolic pathways, 17 pathways of which were significantly enriched. Among these KEGG pathways, three were the most significantly enriched: ‘two-component system’ (map02020), ‘bacterial secretion system’ (map03070), and ‘cationic antimicrobial peptide resistance’ (map01503) (Fig. S4).

In this study, 17 classic types of xenobiotic biodegradation pathways were identified in the metagenomics datasets. Of these, the following pathways contained the largest number of up-regulated genes (Fig. 4c): ‘degradation of aromatic compounds’ (77 unigenes), ‘benzoate degradation’ (72 unigenes), ‘aminobenzoate degradation’ (42 unigenes), ‘chloroalkane and chloroalkene degradation’ (39 unigenes), and ‘nitrotoluene degradation’ (35 unigenes).

### Expression analysis of the genes involved in nitrogen and sulfur metabolism

**Figure 5:**
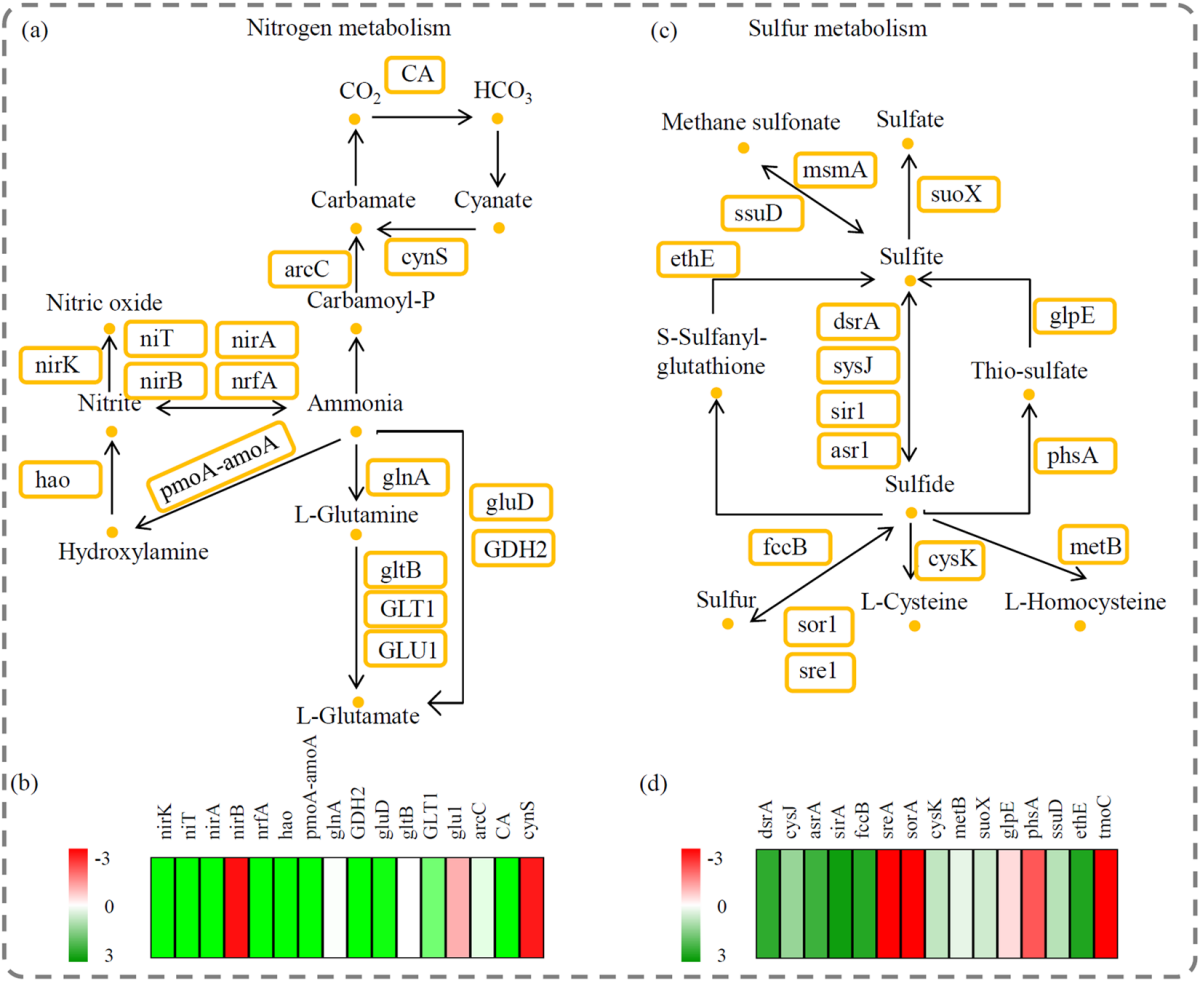
Analysis of the genes involved in the nitrogen metabolic and sulfur metabolic pathways. (**a**) Overview of the nitrogen metabolic pathway. (**b**) The relative abundances of the genes involved in the nitrogen metabolic pathway. (**b**) Overview of the sulfur metabolic pathway. (**b**) The relative abundances of the genes involved in the sulfur metabolic pathway. Red indicated up-regulated and green indicated down-regulated genes. The heatmap scale ranges from −3 to +3 on a log_2_ scale.

H_2_S and NH_3_ are major components in odorous gases^27^. Our study identified a number of key genes involved in metabolizing nitrogen and sulfur; these genes play essential roles in assimilating H_2_S and NH_3_. For the nitrogen metabolism pathway, 16 key genes were identified: *nirK*, *niT*, *nirA*, *nirB*, *nrfA*, *hao*, *pmoA-amoA*, *glnA*, *GDH2*, *gluD*, *gltB*, *GLT1*, *glu1*, *arcC*, *CA*, and *cynS*. Of these genes, nirB, gltB, glu1 and cynS were up-regulated after treatment (Fig. 5a). For the sulfur metabolism pathway, 15 key genes were identified: *dsrA*, *cysJ*, *asrA*, *sirA*, *fccB*, *sreA*, *sorA*, *cysK*, *metB*, *suoX*, *glpE*, *phsA*, *ssuD*, *ethE*, and *tmoC*. Of these genes, *sreA*, *sorA*, *glpE*, *phsA* and *tmoC* were up-regulated after treatment (Fig. 5b).

## Discussion

Increasing evidence shows that microorganisms play an important role in biodegradation; in addition, their community structures were affected by waste gases^28,29^. The interaction between microorganisms and contaminants is a hot topic in research on biofiltration systems^30^. This study established a commercial scale biofiltration system, integrating bacterial and fungal biofilters. The study then evaluated its performance in removing odorous gases. The data showed the complete removal of H_2_S and over 80% of removal for NH_3_. These outcomes suggest the biofiltration system was highly effective in treating odorous gases (Fig. 1). Our data help illustrate the interaction between microbial ecosystems and odorous gases removal in a biofilter at metabolic levels.

In our study, significant differences in microbial community structures were observed. Due to the high levels of H_2_S and NH_3_, a large number of microbes could not survive in the biofilter after a week period of continuous odor-contaminated airflow. On other hand, the microbes that can utilize the ammonia and sulfite will enriched in the biofilter. Thus, the increased microbes are the target strains for improving the efficiency of biofilter.

At the phylum level, *Proteobacteria* was the largest phylum identified in both the BT and AT samples (Fig. 3b). Previous studies showed that several strains belonging to Proteobacteria phylum, such as *Vibrio alginolyticus*, *Stenotrophomonas nitritireducens* L2, and *Nitrococcus mobilis*, played important roles in removing ammonia gas^31–33^. Other *Proteobacteria* strains, such as *Thiomonas* sp.WZW, *Pseudomonas putida*, and *Sulfurimonas denitrificans*, played roles in removing sulfides^15,34,35^.

In our biofilter, the proportions of *Proteobacteria* increased from 38.76% to 78.99% after treatment. This indicated that *Proteobacteria* strains play an important role in degrading H_2_S and NH_3_ containing gases. Further, different strains belonging to *Actinobacteria* and *Firmicutes* phyla have been identified as participating in the degradation of odorous gases^36,37^. For example, *Rhodococcus* sp. and *Arthrobacter* sp., belonging to *Actinobacteria*, were the dominant bacteria in different sulfide and ammonia treatment biofilters^37,38^. Many other strains belonging to *Firmicutes*, including *Moraxella* sp., *Acinetobacter* sp., *Bacillus* sp. and *Exiguobacterium* sp., have been found to be involved in removing ammonia and sulfide^39,40^. In our study, *Actinobacteria* and *Firmicutes* were the top 10 largest phyla after the treatment, suggesting they play roles in degrading odorous gases. Our data provided important cues for isolation of dominant microbes in biofilter after odorous gas treatment.

A previous study found that *Acidithiobacillus thiooxidans* AZ11 is a sulfate-resistant strain and exhibits high sulfur oxidizing activity at low pH conditions^16^. At the genera level, *Acidithiobacillus* (70.1%) was the dominant genus after odorous gas treatment (Table S3). In addition to *Acidithiobacillus*, *Thiomonas* sp. WZW has been enriched and isolated from activated sewage sludge and has been shown to play an important role in enhancing the removal of carbon disulfide^15^. In this study, the genus *Thiomonas* significantly increased (from 0.073% to 0.43%) after treating odorous gases. Based on the high effective removal of NH_3_ in our biofilter, strains belonging to *Acidithiobacillus* and *Thiomonas* genera may play roles in removing ammonia.

NH_3_ and H_2_S are two important indicators for the removal of odorous gases^41,42^. In the past decade, biofilters with different packing media have been widely used to simultaneously remove NH_3_ and H_2_S gases^43^. In our study, most of the genes involved in ammonia and sulfite metabolism were identified in the BT group, and not the AT group. This is consistent with the reduction in microbial community structures in the BT group, compared with the AT group (Fig. 5). A large number of microbes without the capacity in absorption and fixation of NH_3_ and H_2_S could not survive in the biofilter.

Due to the reduction in microbial community structures, the relative abundance of many ammonia metabolism-related genes was decreased in the BT group. Ammonium ion assimilation involves a central metabolic pathway in various plants; glutamine synthetase plays an essential role of incorporating ammonium ions into glutamine and glutamate^44^. Based on different cofactors, glutamine synthetase occurs in three forms: gltB (EC:1.4.1.13, using NADPH), glt1 (EC: 1.4.1.13, using NADH), and glu1 (EC:1.4.7.1, using reduced ferredoxin)^45^. This study identified all three glutamine synthetase forms. There was an increase in the relative abundances of gltB (EC: 1.4.1.13) and glu1 (EC:1.4.7.1). This suggests that gltB and glu1 were the dominant forms of glutamine synthetase when treating odorous gases. NirB is essential for NADH-dependent nitrite reductase activity^46^. The relative abundance of nirB (EC: 1.7.1.15, K00362) was higher in the AT group; this suggests it plays a role in assimilating ammonia-containing odorous gases.

For the sulfur metabolism pathway, five key gene categories were highly expressed in the AT sample group. SreA is an important gene encoding 110 kDa subunit of sulfur oxygenase/reductase; and sorA is another gene encoding 309 amino acid residues of sulfur oxygenase/reductase^47,48^. In this study, there was a higher relative abundance of both *sreA* and *sorA* genes in the AT group compared to the BT group. This suggests there was an activated sulfur oxidation pathway in the biofilter. In addition, *glpE*, encoding a thiosulfate/3-mercaptopyruvate sulfurtransferase; *phsA*, encoding a thiosulfate reductase; and *ethE*, encoding a sulfur dioxygenase, were significantly induced by the odorous gas treatment^49,50^. *Sre1A*, *sorA*, *phsA*, and phsA were involved in sulfur metabolism and were considered to be efficient genes responsible for sulfur fixation in our biofilter.

The performance of biofilters in removing odorous gases was determined based on microbial compositions and metabolic interactions. Based on unigene sequences, significantly differences in microbial community structures were seen between the BT and AT sample groups. At the phylum level, proportions of *Proteobacteria*, *Euryarchaeota*, *Nitrospirae*, and *Parvarchaeota* significantly increased after treatment, indicating their important roles in degrading gases containing H_2_S and NH_3_. Several target strains will be used for improving the efficiency of biofilter by artificial addition. Most genes involved in ammonia and sulfite metabolisms were in the BT group, rather than the AT group. An analysis of the relative abundance identified several key up-regulated genes, which may be the most efficient genes involved in removing odorous gases. Furthermore, efficient genes involved in removing odorous gases will be used for the genetic improvement of target strains. Our findings can be used for improving the efficiency of biofilter and helping the industrial enterprises to reduce the emission of waste gas.

## Supplementary information

Supplementary files

Supplementary files
