# Supplementary material for: Variations in microbial community structure and functional gene expression in bio-treatment processes with odorous pollutants
Source: Sci Rep. 2019 Nov 28;9:17870. doi: 10.1038/s41598-019-54281-0 (PMC6883040; doi:10.1038/s41598-019-54281-0)
Supplement: Supplementary file 1 — Supplementary files [file 41598_2019_54281_MOESM1_ESM.pdf]

Variations in microbial community structure and functional gene expression in bio-treatment processes with odorous pollutants

Weidong Li [1], Shaoqin Cai [2,3], Jianguo Ni [4], Ying Liu [2], Chenjia Shen [2], Huayun Yang [1], Yuquan Chen [2], Jia Tao [2], Yunfeng Yu [2], Qi Liu [1,2,]*

[1] College of Qianjiang, Hangzhou Normal University, Hangzhou 310036, Zhejiang, People's Republic of China;

[2] College of Life and Environmental Science, Hangzhou Normal University, Hangzhou 310036, Zhejiang, People's Republic of China;

[3] College of Environment, Zhejiang University of Technology, Hangzhou 310014, Zhejiang, People's Republic of China;

[4] Hangzhou Ecological Environment Bureau of Xiaoshan Branch, Hangzhou, 311201, Zhejiang, People's Republic of China

* Author for correspondence:

Tel.: +86-571-28867258; Fax: +86-571-28865333.

E-mail address: qiliu@hznu.edu.cn (Q. Liu)

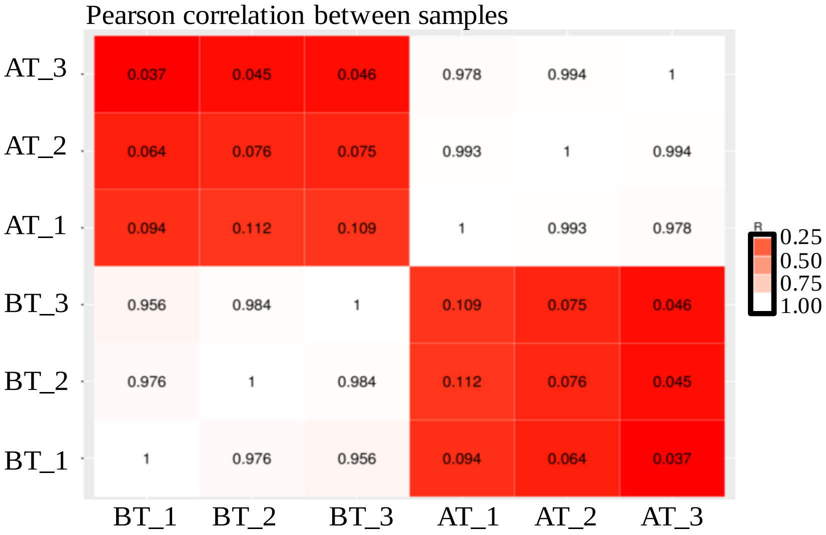

Figure S1 Pair-wise Pearson's correlation coefficients of the three replicates × two sample groups.

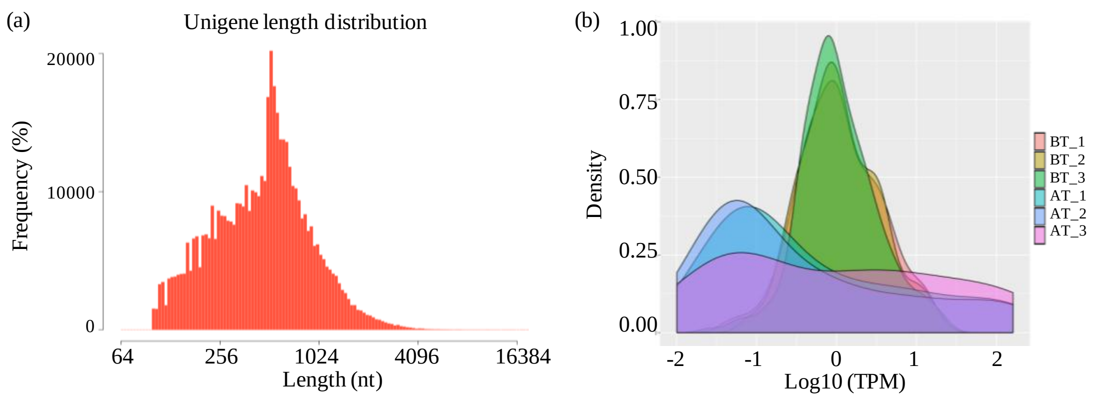

Figure S2 Detail information of the unigenes identified in our study. (a) The length distribution of all predicted unigenes. (b) The densities of unigenes expression in each sample.

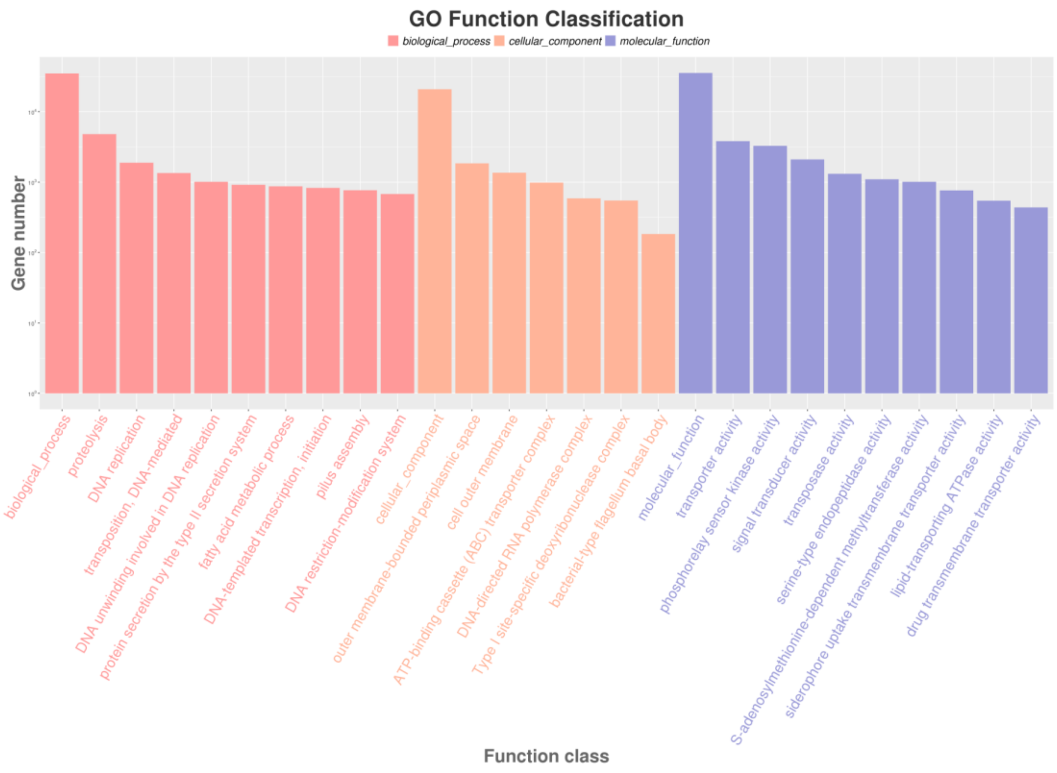

Figure S3 GO classification of all the differential expressed genes.

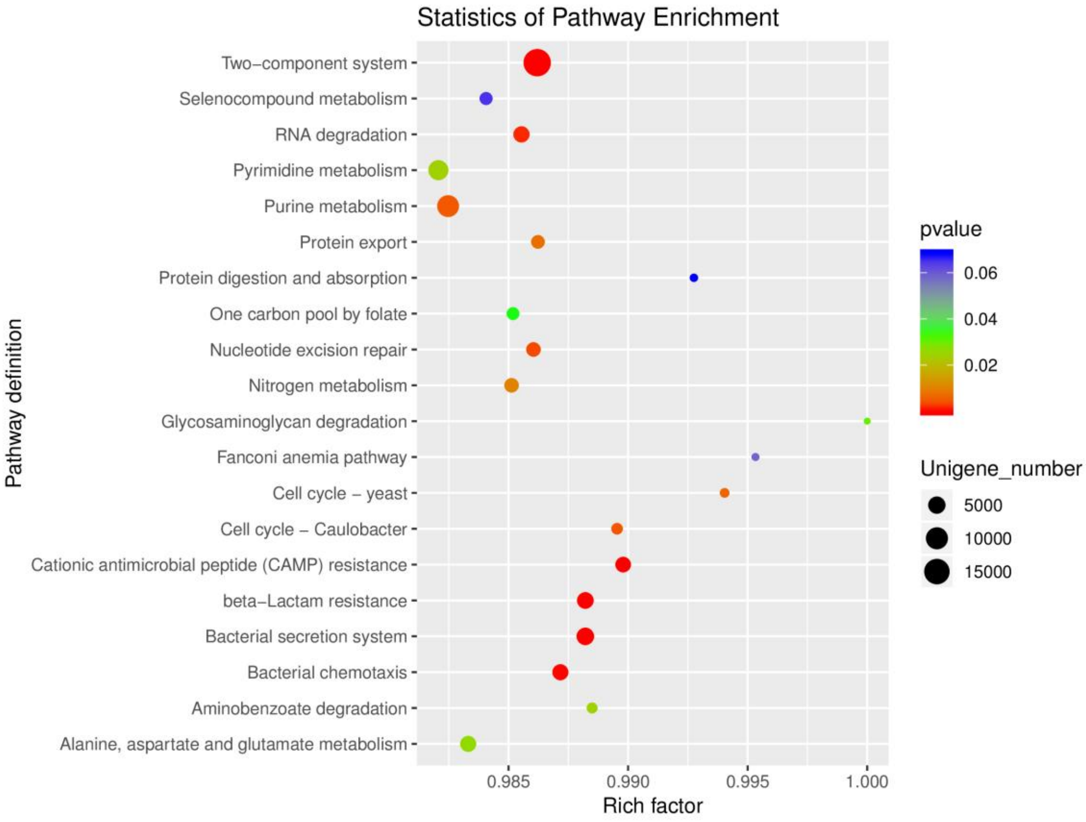

Figure S4 The KEGG metabolic pathways of all the differential expressed genes.
